# Translational Activation of *Oskar* mRNA: Reevaluation of the Role and Importance of a 5' Regulatory Element

**DOI:** 10.1371/journal.pone.0125849

**Published:** 2015-05-04

**Authors:** Matt Kanke, Paul M. Macdonald

**Affiliations:** Department of Molecular Biosciences, Institute for Cellular and Molecular Biology, The University of Texas at Austin, Austin, Texas, United States of America; Emory University, UNITED STATES

## Abstract

Local translation of *oskar (osk*) mRNA at the posterior pole of the Drosophila oocyte is essential for axial patterning of the embryo, and is achieved by a program of translational repression, mRNA localization, and translational activation. Multiple forms of repression are used to prevent Oskar protein from accumulating at sites other than the oocyte posterior. Activation is mediated by several types of *cis*-acting elements, which presumably control different forms of activation. We characterize a 5' element, positioned in the coding region for the Long Osk isoform and in the extended 5' UTR for translation of the Short Osk isoform. This element was previously thought to be essential for *osk* mRNA translation, with a role in posterior-specific release from repression. From our work, which includes assays which separate the effects of mutations on RNA regulatory elements and protein coding capacity, we find that the element is not essential, and conclude that there is no evidence supporting a role for the element only at the posterior of the oocyte. The 5' element has a redundant role, and is only required when Long Osk is not translated from the same mRNA. Mutations in the element do disrupt the anchoring function of Long Osk protein through their effects on the amino acid sequence, a confounding influence on interpretation of previous experiments.

## Introduction

Local translation has emerged as a fundamental mechanism for establishing the subcellular distribution of proteins [1]. A large fraction of all mRNAs appear to exhibit some degree of localization [2,3], and regional differences in mRNA abundance alone can create corresponding differences in protein levels. If the localized mRNAs are subject to translational repression before they are localized, and released from repression after localization, regional differences in protein levels can be greater.

Local translation plays a critical role in early Drosophila development. Specification of the embryonic body plan relies on the actions of a few key mRNAs localized to discrete regions of the oocyte [4]. For anterior/posterior patterning, *bicoid* (*bcd*) mRNA is localized to the anterior margin of the oocyte and the anterior of the embryo, and *oskar* (*osk*) and *nanos* (*nos*) mRNAs are localized to the posterior pole of the oocyte and later the embryo. Each of these mRNAs is translationally regulated [4].

Repression of *osk* mRNA relies on Bruno (Bru), a protein that binds to multiple sites in the *osk* mRNA 3' UTR [5,6]. Multiple other factors are involved in repression, some also binding the mRNA (e.g. Polypyrimidine Tract Binding protein, PTB) [7], some acting in concert with Bru (e.g. Cup) [8,9], one with a role in control of poly(A) tail length (Bicaudal-C) [10,11] and others whose roles are less well defined (e.g. Me31B) [12]. Repression by these various proteins, which act using different mechanisms, must be overcome at the posterior pole of the oocyte. As more than one form of repression appears to be used, it seems likely that more than one form of activation may be required. Several proteins have been implicated in translational activation of *osk* mRNA. One is Orb, which is required to provide *osk* mRNA with a long poly(A) tail [13–15]. For other proteins, including Staufen and Vasa, the specifics of how they promote *osk* mRNA translation remain uncertain [16].

Translational activation of *osk* is dependent on *cis*-acting elements in the *osk* mRNA. Two types of activation elements, the IBEs and certain Bru binding sites, are located in the 3' UTR. The IBEs are short sequences present in multiple copies; mutation of a subset of these eliminates Osk protein production [17]. Although Bru was first identified as a translational repressor, the Bru binding sites positioned close to the 3' end of the mRNA play a second role in translational activation [6]. Mutation of these Bru binding sites causes a substantial decrease in the initial phase of Osk protein accumulation, and largely eliminates the later phase of expression during which the bulk of Osk protein is made [6,18].

A third type of activation element lies in the 5' part of the mRNA, in the region between the two translation initiation codons (both in the same reading frame) used to make Long Osk and Short Osk proteins [19]. These proteins differ only in the amino-terminal extension unique to Long Osk, which is required for cortical anchoring of Osk protein at the posterior of the oocyte [20]. Gunkel et al. concluded that the 5' activation element was essential for *osk* mRNA translation, and that it functions only at the posterior pole of the oocyte and thus could provide the link that coordinates mRNA localization with translational activation [19]. These conclusions had substantial impact. In the context of *osk* regulation, any model for activation of translation would have to incorporate the role of the 5' element. In the broader context of local activation of translation, the *osk* 5' element provided a precedent for activation elements which act only at the site to which the mRNA was localized.

Here we provide a more detailed characterization of the *osk* 5' activation element, with our work leading to conclusions about the importance and role of the element which differ significantly from those published previously. The original work on this element primarily made use of mutants which affected both the Long Osk protein and the *osk* mRNA [19]; potential effects of disrupting Long Osk function were not considered. We confirm the presence of a translational activation element, and more precisely map its location. Notably, mutation of the activation element also disrupts Long Osk function, leading to loss of cortical Osk anchoring. We show that the element is not normally required for *osk* mRNA translation. Instead, the element contributes only to translation of versions of the *osk* mRNA in which the initiation codon for Long Osk is also mutated. Finally, we explain how the conclusion that the element is only active at the posterior of the oocyte relied on an expectation which more recent experiments reveal to be incorrect. The question of why the 5' activation element becomes essential when expression of Long Osk is prevented is intriguing, but of most immediate significance are the conclusions that the 5' element does not have key properties assigned to it, and does not make a necessary contribution to activation of *osk* mRNA translation.

## Results

### Mapping a regulatory element in the *osk* 5’ region

**Fig 1:**
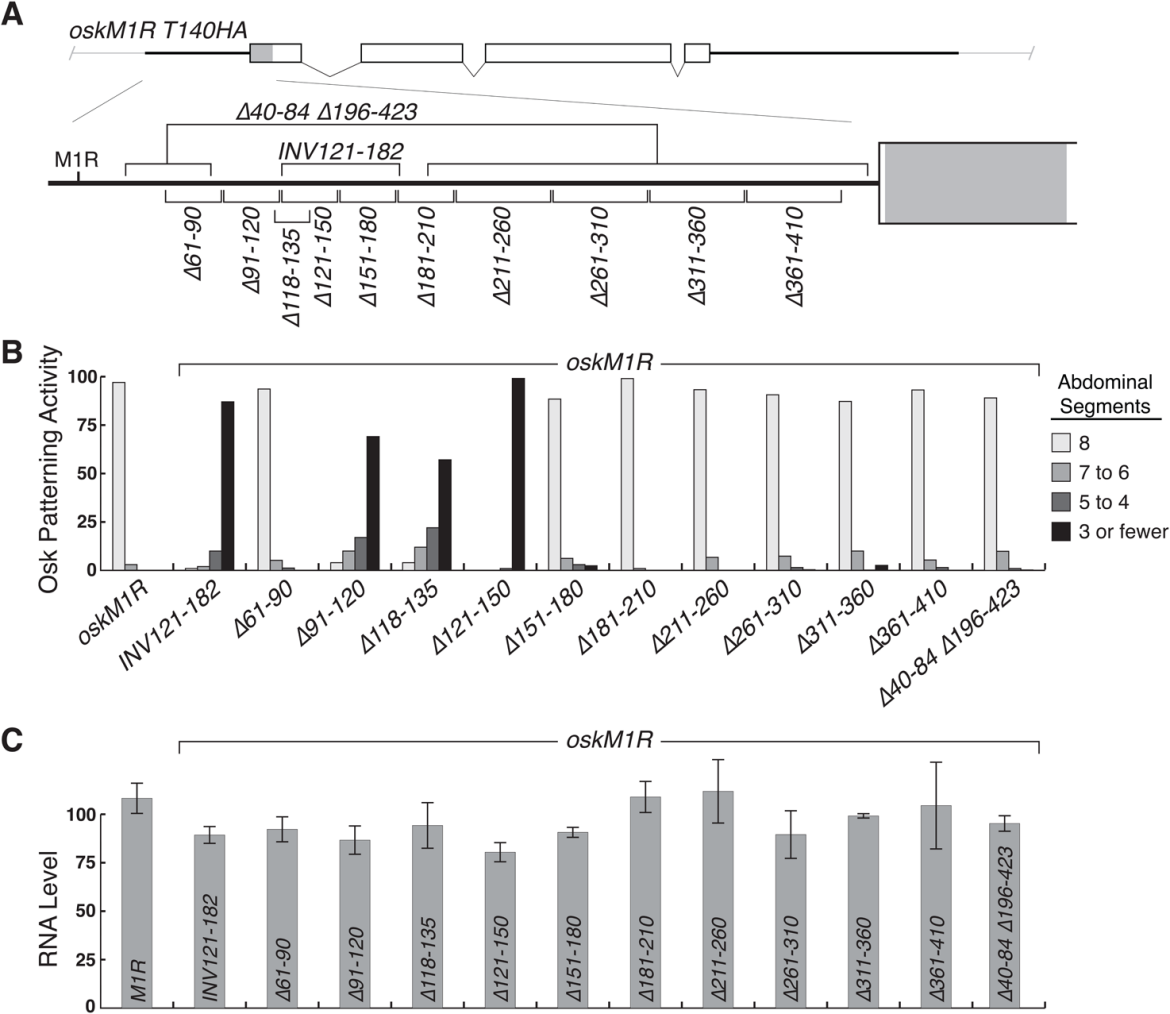
Mapping an RNA element required for *osk* activity. A. Diagram of the 5' region of the *osk* mRNA bearing the M1R mutation, which eliminates translation of Long Osk. The extended 5' UTR is shown as a black line, and the Short Osk coding region as a rectangle. The *oskM1RHA* transgene has the M1R mutation and contains 3 copies of the HA epitope tag (shaded region), inserted after residue T140 (the Short Osk start codon is M139). Deletions are indicated. B. Patterning activity of *osk* transgenes, tested as single copies in the *osk* ^*A87*^ */Df(3R)osk* background (RNA null). The number of abdominal segments corresponds to the level of *osk* activity, with wild type embryos having eight. n values were all over 250, except for *oskM1R INV121-182* (166), *oskM1R ∆61–90* (203), and *oskM1R ∆211–260* (177). No anterior patterning defects were observed for any of the transgenes, including *oskM1R*. C. Levels of *osk* mRNA produced from a single copy of the indicated transgenes. All values are normalized against the level of mRNA from a single copy of the *oskHA* transgene, which is identical to *oskM1RHA* except that it has the wild type M1 codon. Levels of *rp49* were monitored to normalize for amount of RNA used in each assay. Error bars indicate standard error. An analysis of variance (ANOVA) demonstrates no significant difference between RNA levels for any of the transgenes tested (F = 1.066, df = 13, p = 0.417).

To characterize the role in Osk expression of sequences in the 5' portion of the *osk* mRNA, we used transgenes in which the start codon for translation of Long Osk, M1, was mutated (*oskM1R*). Because Long Osk is not produced by *oskM1R* [20], mutations that lie between the start codons for Long Osk and Short Osk will only alter the mRNA sequence, and not the protein coding sequence (Fig 1A). Thus, any change in *osk* activity between *oskM1R* and mutants with lesions in the extended 5' UTR (the sequences upstream from the Short Osk AUG) must be due to a change in *osk* expression, not a structural defect in Osk protein. For these assays, and all those to follow unless noted otherwise, the *osk* transgenes were tested in an *osk* RNA null background and are thus the only source of *osk* mRNA and protein. At least two independent transgenic lines were tested for all transgenes, with similar results. We find that *oskM1R* provides essentially wild type levels of *osk* patterning activity: almost all embryos from mothers expressing only *oskM1R* have normal segmentation (Fig 1B). This is consistent with one previous report [21]. Another study found a low frequency of patterning defects [20], but the sample sizes were small, which could explain the difference.

A 62 nt inversion, similar to that used previously with an *osk*::*lacZ* reporter transgene to disrupt the 5' activation element [19], was introduced into *oskM1R* to make *oskM1R INV121-182*. The mutant had dramatically reduced *osk* patterning activity (Fig 1B). These results confirmed the presence of an element required for translation of the transgene mRNA, and provisionally mapped it to the region affected by the inversion. The inversion could directly alter a regulatory element, by a change in the sequence. Alternatively, the inversion could have an indirect effect, with a change in mRNA structure affecting a regulatory element in nearby sequences. To distinguish between these options, and to test other parts of the *osk* 5' region for a role in translation, an initial series of 30 or 50 bp deletion mutants which systematically remove most of the extended 5' UTR was tested (Fig 1A). Most of the mutants retained normal levels of *osk* patterning activity. Only *oskM1R ∆91–120* and *oskM1R ∆121–150* from these initial mutants were defective, with extremely low levels of *osk* patterning activity (Fig 1B). For these mutants, as well as *oskM1R INV121-182*, mRNA levels appeared to be modestly reduced relative to *oskM1R* (although statistical analysis indicates no significant differences among any of the mRNAs tested; Fig 1C). While a possible reduction in mRNA levels might contribute to the low *osk* activity of these mutants, it cannot be a major cause: *oskM1R INV121-182* is present at a level similar to that of *oskM1R ∆151–180*, which despite the reduced mRNA level retains a high level of *osk* patterning activity. Furthermore, an additional mutant that was not part of the original set (*M1R ∆118–135*; below) also had very low *osk* activity yet had mRNA levels similar to several of the deletion mutants with strong *osk* activity (Fig 1B and 1C).

These results indicated that the *osk* 5' regulatory element resides in the region from nucleotides 91–150 of the *osk* mRNA. Because deletions of much of the remainder of the extended *osk* 5' UTR did not affect *osk* activity, there appear to be no other required regulatory elements. Alternatively, additional elements might act redundantly and therefore would not be detected. To address that possibility, a further transgene was tested. The *oskM1R ∆40–84 ∆196–423* mutant retains the 91–150 region and flanking sequences, but lacks most of the rest of the extended *osk* 5' UTR (Fig 1A). Despite the extensive deletions, the mutant had a level of *osk* patterning activity similar to that of *oskM1R* (Fig 1B). Thus, within the region tested there is only a single RNA segment with regulatory information important for expression of the *oskM1R* mRNA.

**Fig 2:**
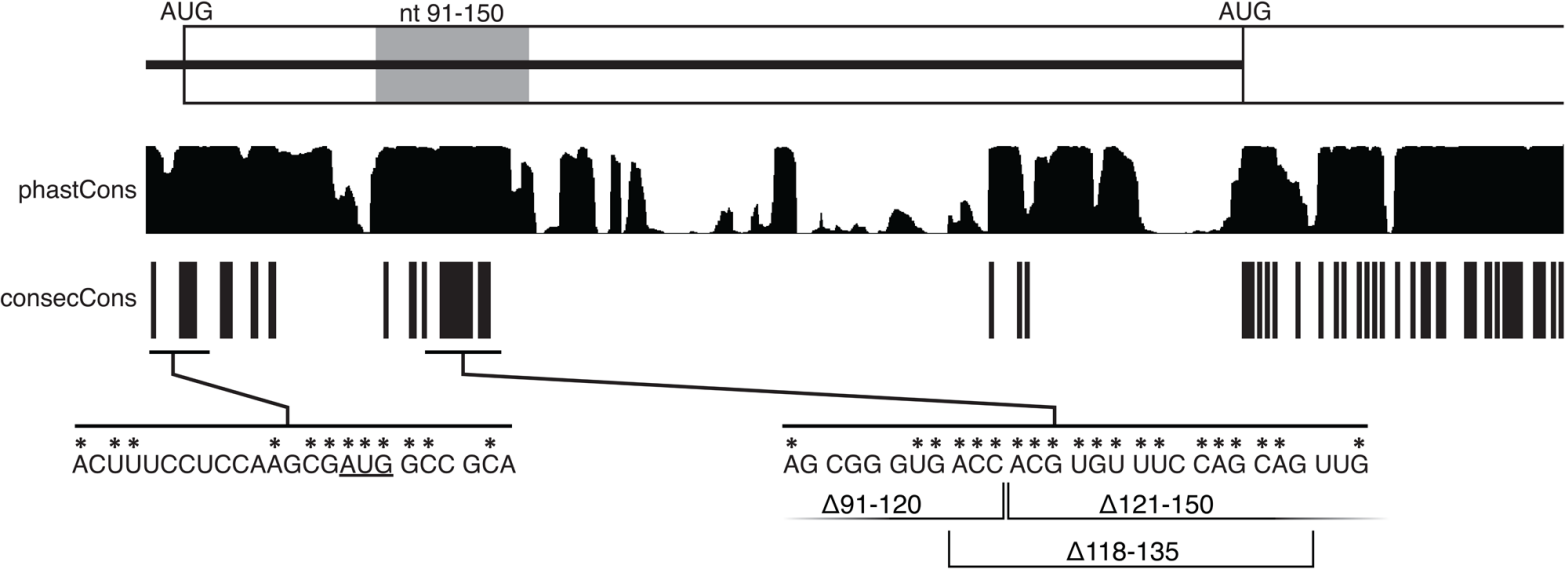
Sequence conservation in the 5' region of the *osk* gene. The diagram at top shows the 5' region of *osk*, with the extended 5' UTR as a black line and the *osk* coding region as a rectangle. The AUG start codons for Long and Short Osk are shown, and the region containing the 5' activation element is shaded. The two analyses of conservation are shown below, with the phastCons output above and the consecCons output below, as indicated. For the latter, each vertical line indicates the presence of 2 consecutive positions that are perfectly conserved among the species analyzed (Methods and Materials). At bottom are segments of the *osk* sequence showing the short regions most highly conserved in the extended 5' UTR. Within the coding region, codons are indicated by spacing, and perfectly conserved positions are identified with asterisks. Endpoints of the indicated deletion mutations are marked.

Although the 5' regulatory element could occupy much of the defined 60 nt region, it could also be contained in a more compact region overlapping the junction of the ∆91–120 and ∆121–150 deletions. Because the regulatory element is expected to be conserved in evolution, we compared the relevant sequences from a number of sequenced *Drosophila* species. In one approach, conservation was assessed by phastCons, which computes conservation scores for aligned sequences based on several criteria: phylogeny, a model of the nucleotide substitution process, and a propensity for conservation to be similar at adjacent sites along the genome [22]. With the default settings of the UCSC genome browser, almost the entire *osk* 91–150 region is shown to be highly conserved, as is a more 5' region and various shorter segments of the extended *osk* 5' UTR (Fig 2). While this might suggest that most of the 91–150 region is highly conserved and potentially part of the regulatory element, the conservation may be overstated. phastCons scores short, highly-conserved regions similarly to long, moderately conserved regions. Additionally, gaps in the alignment are treated as missing data, which may overestimate conservation. As an alternate approach, we surveyed the same region for clusters of highly conserved sequences, a characteristic that might be expected for an RNA regulatory element (consecCons; Materials and methods). This screening was more stringent, with many fewer regions of high conservation identified. Notably, the longest stretch of highly conserved nucleotides lies at the junction of the ∆91–120 and ∆121–150 deletions. Within the conserved region, it appears that the exact sequence is conserved, not simply the protein coding sequence. If maintaining the Long Osk protein sequence provided the only selective pressure, some DNA sequence variation would be allowed for use of alternate codons; this is not the case (Fig 2).

A mutant lacking just the highly conserved sequences, *oskM1R Δ118–135*, had dramatically reduced *osk* patterning activity (Fig 1B), but not mRNA level (Fig 1C). Thus, the highly conserved sequences are critical for function of the 5' regulatory element.

### The *osk* 5' regulatory element activates translation

**Fig 3:**
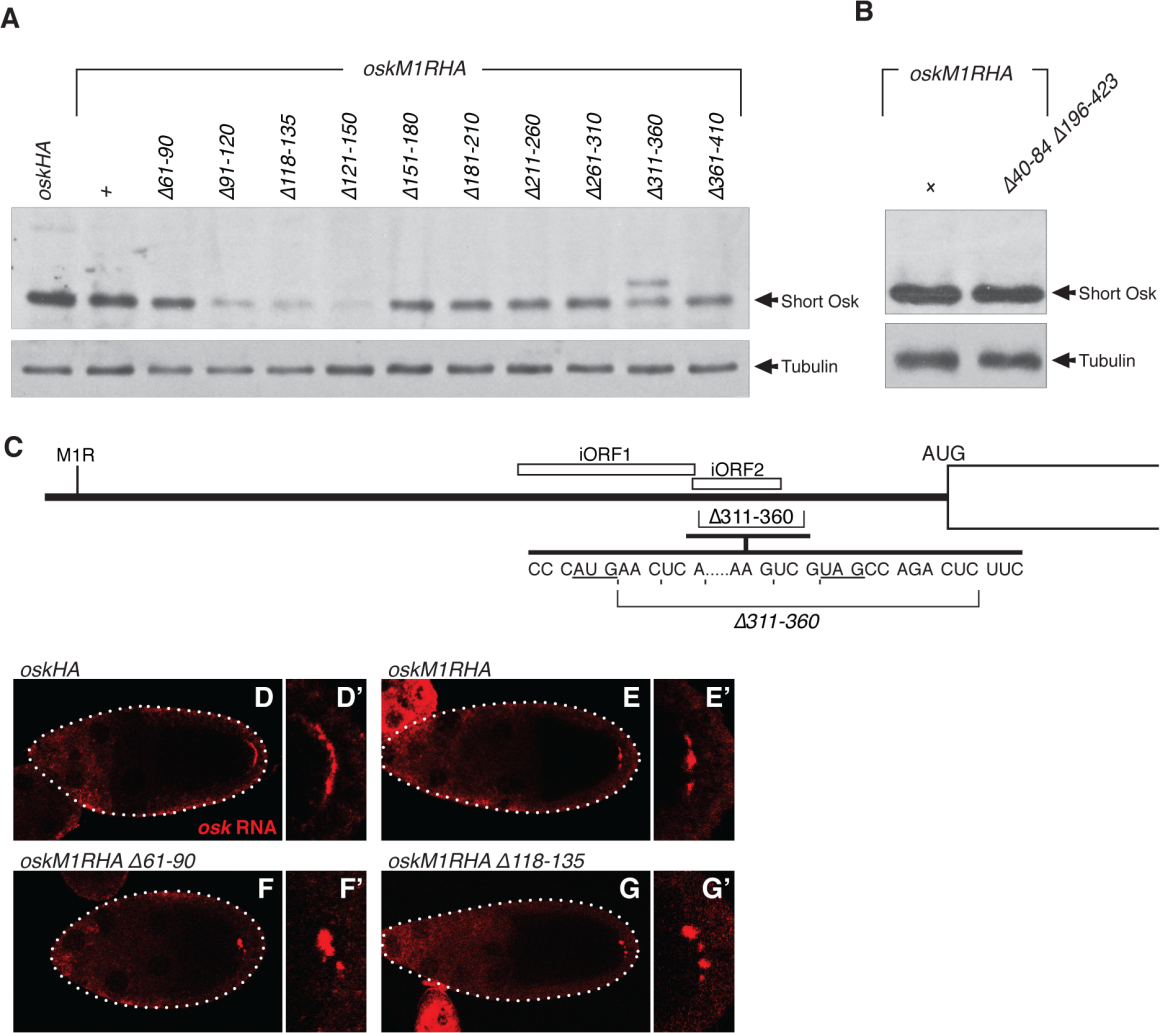
The 5' element is required for translational activation. A and B. Western blot analysis of transgenes expressed as single copies in the *osk* ^*A87*^ */Df(3R)osk* background. Tubulin is detected as a loading control. C. Diagram of the *oskM1R* 5' region, showing the positions of the two iORFs and how the ∆311–360 deletion fuses iORF2 to the Osk reading frame, and thus can produce the novel protein band detected in A. The partial sequence shown has the reading frame for Osk protein indicated by spaces, and the reading frame for iORF2 indicated by vertical hash marks. D-G. In situ hybridization to detect transgene mRNAs in the *osk* ^*A87*^ */Df(3R)osk* background (panels D'-G' are magnified views of the posterior region to better show the mRNA distributions). For the *oskHA* transgene, which makes both Long and Short Osk, the mRNA is tightly restricted to a posterior crescent (D,D'). The *oskM1R* transgene lacks Long Osk and its anchoring function, and the mRNA has a more punctate distribution (E,E'). Similarly, both of the mutants tested, one with normal *osk* activity (F,F'; the ∆61–90 deletion) and one largely lacking *osk* activity (G,G'; the ∆118–135 deletion), have the same punctate distribution of mRNA.

In addition to scoring the transgenes for *osk* body patterning activity, we more directly tested protein levels by western blot analysis of ovarian proteins. The strong embryonic patterning defects from deletion of *osk* mRNA sequences 91–120, 121–150 and 118–135 in *oskM1R* were accompanied by correspondingly low Short Osk protein levels (Fig 3A). By contrast, the mutant retaining the critical regions but lacking much of the rest of the extended *osk* 5' UTR (*oskM1R ∆40–84 ∆196–423*) had a high level of Short Osk (Fig 3B).

Before considering why protein levels are reduced, it is noteworthy that one mutant—*oskM1R ∆311–360*—produced two proteins, one the size of Short Osk, and one larger. Contained between the initiator codons for Long and Short Osk are two additional AUG codons, not in the Long/Short Osk reading frame. Each is followed by a short open reading frame (internal ORF, or iORF; 27 or 13 codons, respectively) and a stop codon. The ∆311–360 deletion removes the stop codon for iORF2, and shifts the iORF2 reading frame to that normally used for Long and Short Osk (Fig 3C). The size of the novel protein is consistent with use of this AUG for initiation of translation of a novel protein, fusing the start of iORF2 to the Osk open reading frame. This strongly suggested that at least one, and possibly both, of the short iORFs is normally translated. How initiation of Short Osk translation occurs is unknown, and could involve scanning of preinitiation complexes formed at the mRNA cap, or a form of internal ribosome entry. Knowing that an intermediate AUG, positioned upstream of the AUG for Short Osk, is used to initiate translation may be useful in elucidating the mechanism of Short Osk translational initiation.

The protein accumulation defects of the *oskM1R* 5' mutants must result from inefficient translation, as mRNA levels were close to normal, and the mutations do not affect the encoded proteins. However, because posterior localization of *osk* mRNA is normally required for Osk protein accumulation, a defect in *osk* mRNA localization can indirectly disrupt translation. We examined the mRNA distribution of the *oskM1R ∆118–135* mutant, using *oskM1R* for comparison. As for the assays of *osk* activity (above), these experiments were performed in *osk* RNA null flies lacking any other source of *osk* mRNA. Consequently, there is no possibility that an mRNA localization defect would be masked by piggybacking, the phenomenon in which localization-defective *osk* transcripts co-localize with wild type *osk* transcripts [23]. In the absence of Long Osk, which provides an anchoring function, *oskM1R* transcripts were not as tightly restricted to the posterior pole of the oocyte as normal (Fig 3E). A similar distribution was found for each of the mutants tested, including examples which retain (*oskM1R ∆61–90*)(Fig 3F) or lack (*oskM1R ∆118–135*)(Fig 3G) the 5' element. Thus, a defect in mRNA localization was not responsible for the reduced protein level of the *oskM1R ∆118–135* mutant, and translation must have been disrupted.

**Fig 4:**
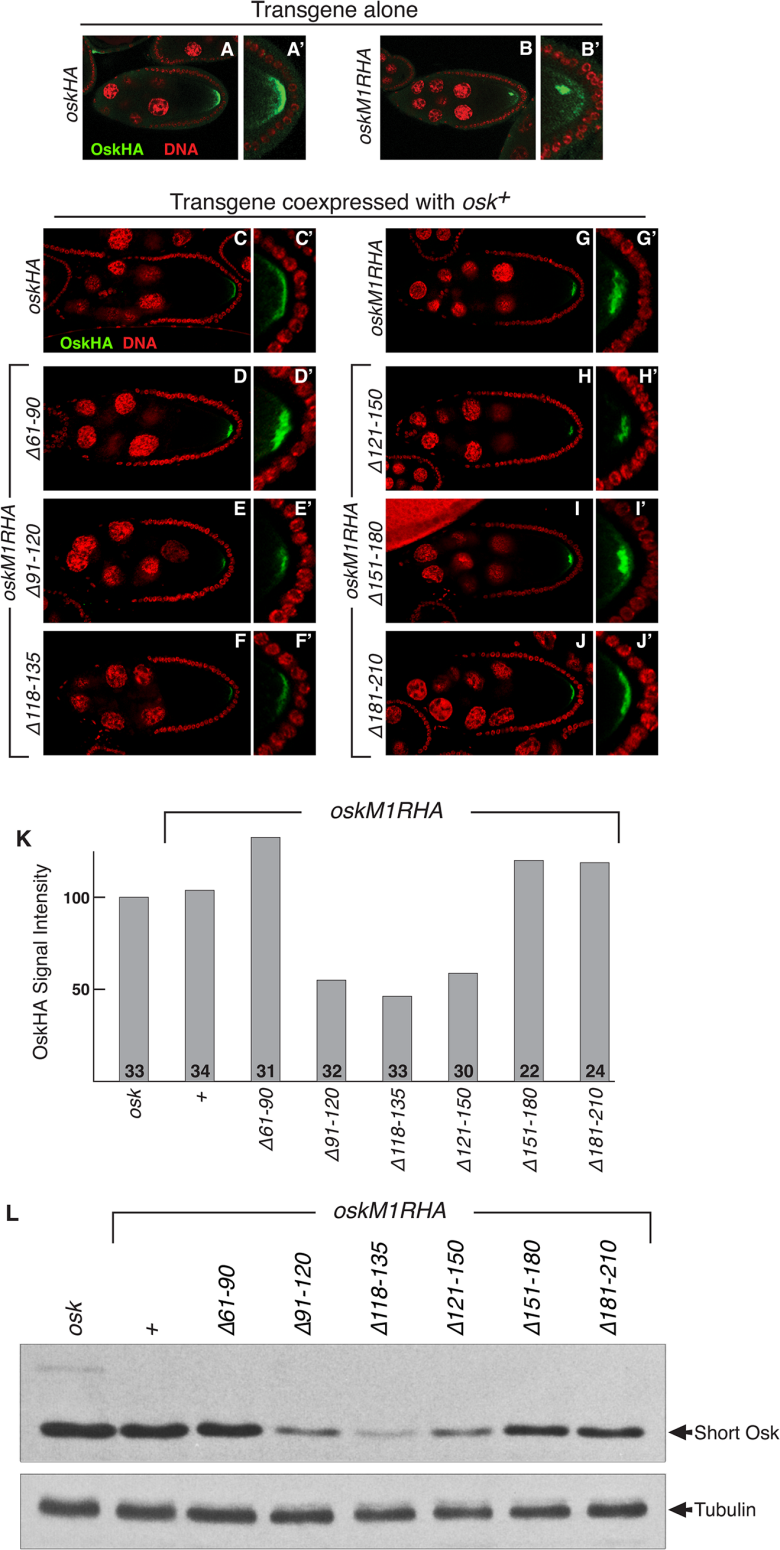
The 5' element is required for the early phase of Osk protein accumulation. A-B,A'-B'. Detection of transgenic OskHA protein expressed from single copies of the indicated transgenes in the *osk* ^*A87*^ */Df(3R)osk* background (RNA null). Panels A'-B' are magnified views of the posterior of the oocyte to better show the proteins. Green is OskHA and red is DNA detected with ToPro-3. C-J,C'-J'. Detection of transgenic Short OskHA protein expressed from single copies of the indicated transgenes in the presence of endogenous Long Osk for anchoring. Panels C'-J' are magnified views of the posterior of the oocyte to better show the proteins. I. Quantification of protein levels from the imaging experiments of C-J. OskHA signal intensities (Methods and Materials) are shown normalized to that from the *oskHA* transgene. The number of oocytes scored is indicated at the bottom of each bar. Error bars indicate standard error. J. Western blot analysis of transgenes expressed as single copies in the presence of a wild type copy of *osk*. Only the transgenic Osk protein is detected using anti-HA antibodies. Tubulin is detected as a loading control.

Although the western blot analysis revealed defects in Osk protein accumulation for the 5' region mutants, this represents an average of Osk throughout all stages of oogenesis because the samples were entire ovaries. The accumulation of Osk during oogenesis begins at stage 9, but the bulk of Osk protein is made late [18]. Consequently, there may be different forms of translational activation that operate at different stages. Indeed, mutations in the *osk* 3' UTR C region BREs most severely disrupt the later phase of Osk accumulation [6]. To determine if the 5' region mutants disrupted the early phase of translation, proteins were monitored by immunodetection and confocal microscopy (this approach cannot be used to monitor protein levels at later stages of oogenesis, because deposition of the vitelline membrane greatly reduces accessibility for antibodies). Initial experiments were performed, as above, with transgenes expressed in an *osk* RNA null background (Fig 4A and 4B). Because Long Osk was not provided by the *oskM1R*-based transgenes, Short Osk protein was not properly anchored at the posterior cortical region and appeared in puncta which were often displaced from the cortex (Fig 4B). This presented a challenge for quantitation, as the puncta were irregularly distributed in multiple focal planes. As an alternate approach, we tested the transgenes (all of which include a 3xHA epitope tag for detection) in an *osk+* background. In this situation endogenous Long Osk anchors the transgenic epitope-tagged Osk protein to the cortex. The transgenic protein alone is detected with anti-HA antibodies (Fig 4C–4J).

Using this assay, we found that the *oskM1R* 5' region mutants with normal patterning activity produced Osk protein at levels similar to *oskM1R* (Fig 4D, 4I, 4J and 4K). The mutants with greatly reduced patterning activity showed a clear reduction in protein levels at stage 9 (Fig 4E, 4F, 4H and 4K). By image analysis the *oskM1R ∆91–120*, *oskM1R ∆118–135* and *oskM1R ∆121–150* mutants had roughly half the normal level of Osk at stage 9 of oogenesis (Fig 4K). The western analysis indicated a larger reduction for each (Fig 3), although we have not quantitated the blots.

One explanation for why different amounts of Osk were detected in the imaging and western assays is that the presence of endogenous Osk in the imaging experiments could have enhanced translation of the *oskM1R*-based mutants (although there is no enhancement of *oskM1R*). If so, western blot analysis comparing the mutants expressed either alone or with endogenous *osk* should reveal a difference in the amount of protein made by the mutants. We repeated the western blot analysis, now with the transgenes in an *osk+* background (Fig 4L). Comparison of transgenic Osk protein levels with (Fig 4L) or without (Fig 3A) endogenous Osk leads to two conclusions. First, in both western blot assays the reduction in Osk protein levels for the defective mutant appears to be greater than obtained by the confocal imaging assay. Second, the presence of endogenous Osk does, nevertheless, appear to slightly increase Osk production from the transgenes, consistent with a feedback loop stimulating Osk production [24]. While endogenous Osk does appear to enhance transgenic Osk expression, this cannot fully account for the differences observed between the confocal imaging and western blot assays; other factors which could contribute to the differences are considered in the Discussion.

Despite the uncertainty about how much each option contributes to the difference in the transgenic Osk protein levels determined in the two assays, our results clearly show that mutation of the *osk* 5' region translational activation element reduces but does not eliminate translation for the initial accumulation of Osk protein. There must also be an effect of the mutations on the late phase of Osk accumulation, but whether this is more severe than at the early stages cannot be definitively established from our results.

### The *osk* 5' activation element is normally dispensable

**Fig 5:**
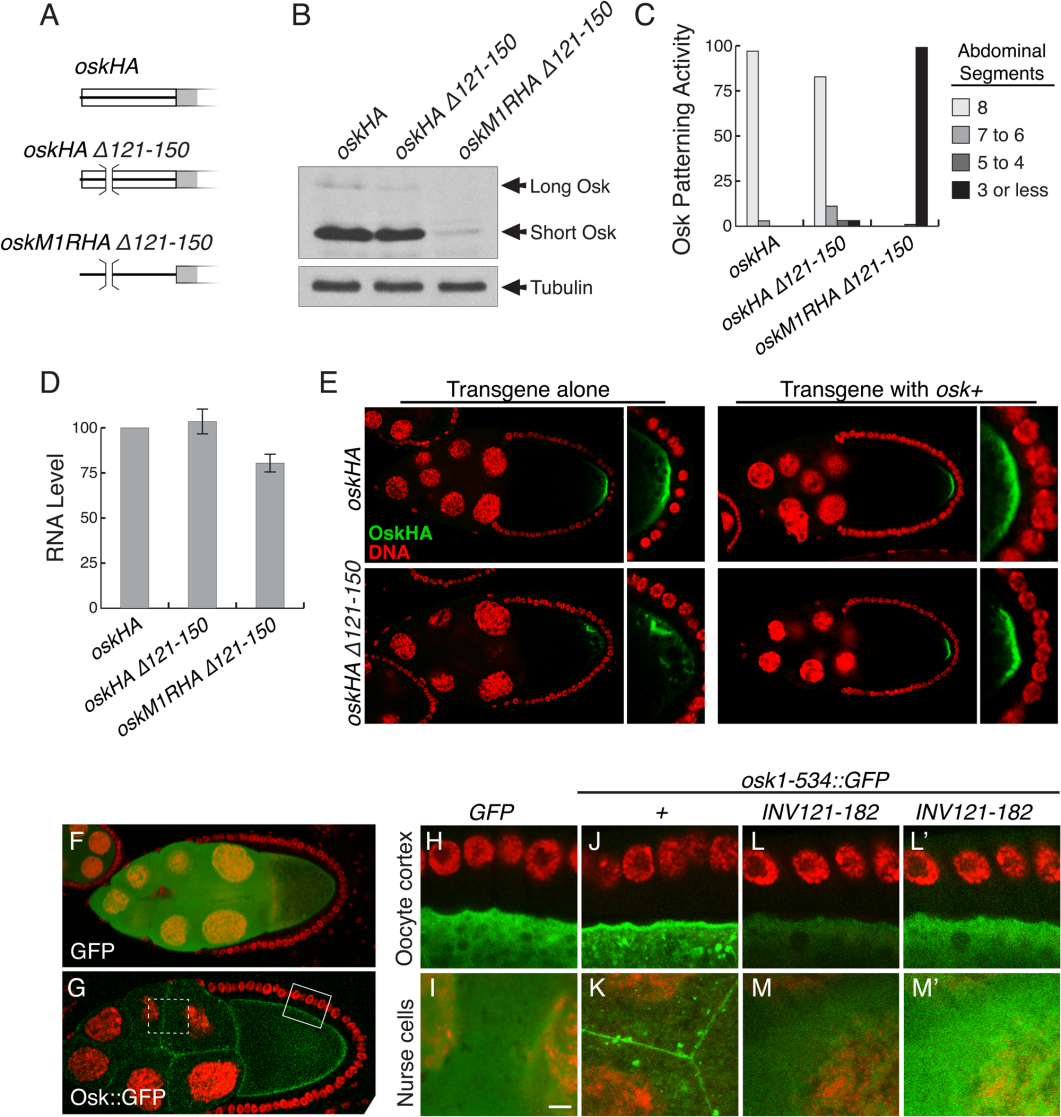
Effects of mutating the 5' element on translation and Long Osk function. A. Diagram of the 5' region of the transgene mRNAs, using the conventions from Figs 1 and 2 but with the deletions marked by brackets and gaps. B. Western blot analysis of transgenes expressed as single copies in the *osk* ^*A87*^ */Df(3R)osk* background. C. Patterning activity of *osk* transgenes, tested as single copies in the *osk* ^*A87*^ */Df(3R)osk* background (RNA null). The number of abdominal segments corresponds to the level of *osk* activity, with wild type embryos having eight. n values were *oskHA*, 511; *oskHA ∆121–150*, 260; *oskM1RHA ∆121–150*, 324. D. Levels of *osk* mRNA produced from a single copy of the indicated transgenes. All values are normalized against the level of mRNA from a single copy of the *oskHA* transgene. Levels of *rp49* were monitored to normalize for amount of RNA used in each assay. Error bars indicate standard error. E. Detection of transgenic OskHA expressed from single copies of the indicated transgenes. For the panels at left, the transgenes were tested in the *osk* ^*A87*^ */Df(3R)osk* background, revealing the anchoring defect of the OskHA∆121–150 mutant, which lacks aa 36–45. This defect is rescued when coexpressed with wild type Long Osk, as shown in the panels at right. F-G. The amino terminal domain of Osk confers anchoring on GFP. F shows the distribution of GFP, and G shows the distribution of the Osk::GFP fusion protein from transgene *UAS-osk1-534*::*GFP*, which includes the first 534 bp of the *osk* mRNA and encodes a fusion protein with the first 173 amino acids of Osk, including the entire Long Osk amino terminal domain. The fusion protein is highly enriched at the oocyte cortex and at nurse cell boundaries. White boxes outline the types of regions shown in panels H,J,L (solid lines) and I,K,M (dashed lines), although these are not the same egg chambers as in those panels. Green is GFP (or Osk::GFP), red is DNA (nuclei) stained with ToPro-3. H-M. Detection of Osk::GFP fusion proteins in stage 10 egg chambers. All panels are at higher magnification than in F and G to highlight anchoring at the oocyte cortex (H,J,L) or at nurse cell boundaries (I,K,M)(the scale bar is 5 μm). For panels I, K, M and M' the images show a portions of several nurse cells and the boundaries between them. Signal intensities can only be compared between panels J-M, which were imaged under identical conditions. Panels L' and M' are identical to L and M except that the green signal was enhanced to better show the absence of any anchoring. The level of protein from the *UAS-GFP* transgene is much higher than from the *UAS-osk1-534*::*GFP* transgenes, and lower intensity laser settings were used to obtain images in F, H and I with signal intensity comparable to G, J and K. Anchoring of the Osk::GFP protein is manifested in the enrichment at the cortex, along nurse cell boundaries, and the punctate appearance in the cytoplasm. Neither GFP alone nor the Osk INV121-182::GFP protein shows any similar anchoring. Green is GFP (or Osk::GFP), red is DNA stained with ToPro-3.

Thus far, our characterization of the 5’ activation element has been performed in the context of *oskM1R*, so as to eliminate any effect of the mutations on the function of Long Osk protein. To extend our analysis, we also constructed the *osk ∆121–150* mutant (which retains wild type M1 and will thus make a mutant form of Long Osk, in addition to wild type Short Osk) (Fig 5A). We recognized that any effect on Long Osk levels might be due to either a change in translation (from loss of the activation element) or a change in protein stability (from alteration of Long Osk structure). Nevertheless, determining if there was a change is Long Osk protein levels was a necessary first step in understanding the cause of any such change.

The *osk ∆121–150* mutant was compared to an *osk+* transgene, and to the *oskM1R ∆121–150* mutant, all tested in the *osk* RNA null background (and all carrying the HA epitope tag). Surprisingly, the consequences of the ∆121–150 mutation varied dramatically, depending on whether *oskM1* was wild type or mutant. If the Long Osk start codon was not mutated, deletion of the *osk* 121–150 sequences had only minor effects: *osk* patterning activity was strong (Fig 5C), and the levels of Short and Long Osk proteins were only slightly reduced (Fig 5B). In sharp contrast, the same ∆121–150 deletion when the Long Osk start codon was mutated largely eliminated *osk* patterning activity (Fig 5C), and the level of Short Osk was very low (Fig 5B). Why mutation of the 5' activation element should have had such different consequences for *osk* and *oskM1R* is not clear. There are differences in mRNA levels, but as shown above such differences cannot explain the dramatic defects of the *oskM1R ∆121–150* mutant. It cannot simply be that the presence of Long Osk protein suppressed the ∆121–150 regulatory defect, as the reduced level of Short Osk from *oskM1R ∆121–150* persisted in the presence of a source of Long Osk (above). We consider possible explanations in the Discussion.

Although deleting nt 121–150 when the Long Osk start codon was intact did not substantially change the level of Long or Short Osk proteins, this mutation (which deletes Long Osk amino acids 36–45) did disrupt the Long Osk anchoring function: Osk protein was displaced from the posterior cortex (Fig 5E). Coexpression with *osk+* corrected this defect (Fig 5E), as expected from provision of anchoring function from wild type Long Osk.

The anchoring defect of *osk ∆121–150*, while readily detectable, did not lead to complete dispersal of Osk protein (Fig 5E). Other portions of the Osk protein engage in assembly of a large RNP structure, the germ plasm, at the posterior of the oocyte [24], and these interactions may be responsible for persistence of the mutant Osk in large puncta. To consider the effect of mutations on anchoring of hybrid proteins which do not include the full Osk protein (such as those used by Gunkel et al.), we also tested versions of a *UAS-osk1-534*::*GFP* transgene [25]. The *UAS-osk1-534*::*GFP* transgene, expressed under GAL4 control, includes the first 173 amino acids of Long Osk and is anchored to the cortical regions of the oocyte and nurse cells, as well as the boundaries between these cells [25](Fig 5G and 5J–5K), while GFP alone is dispersed throughout the cytoplasm (Fig 5F and 5H–5I). When the INV mutation was introduced into this transgene, anchoring was eliminated and the protein was dispersed throughout the nurse cells and oocyte in a pattern indistinguishable from that of GFP alone (Fig 5L and 5M; panels L' and M' have enhanced GFP signal to show the absence of anchoring). The INV mutation also led to a reduction in protein level, but this was much less dramatic than the effect of the mutation on production of Osk from *oskM1R*. It is possible that the INV mutation slightly destabilizes the protein because of the exchange of amino acids 36–56 with those encoded by the inversion. These results demonstrate that for a hybrid protein in which the only region of Osk included is the Long Osk anchoring domain, a mutation used to disrupt the 5' activation element also eliminated anchoring.

## Discussion

The *osk* mRNA has served as a model for understanding the various mechanisms involved in local expression of an mRNA in a restricted region of a cell, in this case the oocyte. A key step in this program of regulation is translational activation of the mRNA following its localization in a repressed state. A long-standing belief about activation of *osk* mRNA was that it relies on an essential regulatory element in the 5' region of the mRNA, and that this element acts specifically and only at the posterior pole of the oocyte. Characterization of this element presents a challenge, as it lies within a region that also encodes the Long Osk protein. Thus, mutations in the element will affect both the mRNA and the encoded protein, an issue not previously considered in interpretation of results. This is not simply a hypothetical concern, as we have shown that mutations which disrupt the RNA element also disrupt Long Osk-mediated protein anchoring, with a complete loss of anchoring for fusion proteins in which the only Osk domain included is the Long Osk anchoring domain.

Our work redefines the roles and properties of the 5' element. Most importantly, we find that key conclusions of the initial characterization are not supported. One conclusion was that the element played an essential role in activation of *osk* mRNA translation [19]. We find that the element is completely dispensable, as long as the translational start codon for Long Osk is intact. The earlier work of [19] tested primarily *osk*::*lacZ* reporter transgenes, which included only the amino terminal portion of Osk and for which a defect in protein anchoring may have affected interpretation of results. Notably, the only *osk* transgene used to assay the effect of deleting the activation element also had a mutation eliminating the Long Osk start codon (their transgene *oskM1L∆1*). The failure of this transgene to provide *osk* activity can now be explained by the combined effects of mutating the Long Osk start codon and deleting the 5' activation element. Thus, both sets of data are in agreement if we consider only *osk* transgenes.

A second key conclusion of [19] was that the activation element was only functional at the posterior pole of the oocyte. The evidence for this conclusion involved an *osk*::*lacZ*::*bicoid* mRNA which was misdirected to the anterior of the oocyte, and failed to be translated there. In this transgene a large portion of the *osk* 3' UTR was removed, with the expectation that these sequences were not required for translation. However, it has since been shown that the missing portion of the *osk* 3' UTR does in fact include sequences essential for translation, the IBEs [17] and the *osk* C region Bru binding sites [6], although the implication of these results on interpretation of the 5' activation element experiments has not been previously noted. We argue that the absence of these elements is fully sufficient to explain the failure of the mislocalized *osk*::*lacZ*::*bicoid* mRNA to be translated. While it remains a formal possibility that the element has a posterior localization-dependent role, there is no evidence to support such a model.

### Role of the 5' regulatory element

With the important caveat that our work only addresses the function of the 5' regulatory element when the Long Osk start codon is mutated, it seems likely that the element performs a redundant function (below) and understanding that function will advance our knowledge of translational regulation. To characterize the role of the 5' regulatory element we used transgenes in which mutations to disrupt the element affected only the mRNA, and not the encoded protein. In this manner we showed that the element functions in translation, not mRNA localization or stability. Our analysis indicates that there is a single regulatory element (or cluster of elements) in the extended *osk* 5' UTR, and strongly suggests that it is contained, in part or whole, in a short, very highly conserved sequence. The interpretation that this work identifies a regulatory element rests on the assumption that the mutations are loss-of-function, and disrupt the element. Alternatively, the defective mutants could be gain-of-function, interfering with *osk* expression because of novel RNA sequences or structures formed by the mutations. The possibility that novel binding sites for disruptive factors have been created seems extremely unlikely: the defective mutants—three different deletions and one inversion—create four different sets of novel sequences; the probability that any one mutant would acquire a novel function is not high, but that all four would acquire the same novel function seems implausible. Predicted folding of wild type and mutant RNAs does not reveal any consistent, substantial change in stability, and so that possibility also appears very unlikely. The high conservation of sequences in the region implicated in regulation also argues that the mutations have defects because of loss of an element, although this argument is tempered by the fact that the same region must be conserved for the function of Long Osk protein.

In our experiments to monitor the 5' activation element, loss of the element appeared to have a larger effect in the imaging assay than in the western assay (which was not quantitated). The presence of endogenous Osk in the imaging assays (in which HA-tagged transgenic Osk was monitored) is expected to enhance production of Osk from the mutant transgene [24], and could partially explain the apparent difference. There are two other likely contributions. First, we know that the image analysis underestimates the degree to which posterior Osk protein levels are reduced (see Materials and Methods), but this may not account for all of the difference. Second, it is possible that the *osk* 5' region mutations more strongly disrupt the late phase of *osk* translation, just as observed for the *osk* C region BRE- mutants [6]. If so, the western analysis, which includes late stage egg chambers, would show a stronger defect, consistent with what we have observed.

A puzzling aspect of our results is why the 5' activation element is dispensable unless the translation start codon for Long Osk is mutated. Mutating the Long Osk start codon has two direct consequences: loss of the Long Osk protein, and loss of assembled ribosomes moving along the mRNA towards (and through) the start codon for Short Osk. Presumably, one or both of these features can substitute for the 5' element. The simple presence of Long Osk is not sufficient, as coexpression of Long Osk fails to rescue the translation defect of the 5' element mutants. A more arcane version of a requirement for Long Osk is that the nascent protein must be provided in *cis*, being able to compensate for loss of the 5' activation element only on the RNA from which it is translated.

Another option is that the element acts redundantly with transiting ribosomes for translation from the Short Osk start codon. Assuming that translation of both Long and Short Osk relies on scanning initiation, in which preinitiation complexes assemble at the 5' cap and move along mRNA, then both ribosomes and preinitiation complexes will transit along the mRNA towards the Short Osk initiation codon. For the *oskM1R* mutant mRNA, only preinitiation complexes would be engaged. RNA secondary structures impede movement of the preinitiation complex [26], and helicase activity is required to unwind these obstacles [27]. By contrast, the ribosome has an intrinsic mRNA helicase activity, which can disrupt very stable downstream helices [28]. An explanation of our results is that there are barriers to 48S progress in the *osk* extended 5' UTR, either bound factors or RNA secondary structures (although no strongly stable structures are predicted), and these must be removed for translation of Short Osk. The ribosome would be able to perform this function, allowing a trailing preinitiation complex to proceed to the start codon for Short Osk. By contrast, a preinitiation complex by itself would be unable to efficiently do so. In this model the 5' element could act by recruiting a helicase to assist the preinitiation complex. It is not certain that translation of Short Osk relies on a cap-dependent scanning mechanism, but even with a form of internal initiation the same principle could apply: initiation requires the removal of an interfering factor, and is achieved either by a ribosome making Long Osk, or by a factor recruited by the 5' activation element.

Progress in understanding the function of the 5' element will require identification of the factor it binds. We have not yet detected any protein that binds specifically to RNAs containing the element. [19] described proteins that bind to a broader region containing the element. However, there is not a good correlation between regions showing strong binding and regions we have shown to be important for activation, and so these proteins are not strong candidates for activators.

## Materials and Methods

### Fly Stocks and Transgenes

*w* ^*1118*^ flies were used as wild-type. *Df(3R)osk* [6] and *oskA87* were used for all *osk* rescue experiments. Genomic *osk* transgenes included a 3xHA epitope tag, inserted after amino acid 140, to facilitate western blot analysis and, in some cases, to distinguish the protein from endogenous Osk [25](J Jones and PMM, submitted). This tag does not detectably alter *osk* expression or activity. The deletion and inversion mutations were constructed using PCR, and introduced into the *osk* rescuing transgene [29] by standard methods. For each transgene, at least two independent transgenic lines were used for analysis. The *UAS-osk1-534*::*GFP* transgene [25] was modified in the same manner. The version of GFP in this transgene is mGFP6 [30].

### Cuticle analysis

Cuticle preparations [31] were mounted in Hoyer’s Mounting Medium and viewed with a Nikon Eclipse E600 microscope.

### Determination of sequence conservation

Sequence conservation across the 5’ portion of *osk* was assessed on the UCSC Genome Browser based on the conservation scores calculated by phastCons [22]. phastCons scores short, highly-conserved regions similarly to long, moderately conserved regions. Additionally, gaps in the alignment are treated as missing data, which may overestimate conservation. As another measure of conservation, the 5’ portions of *osk* from 11 *Drosophila* species (*D*. *ananassae*, *D*. *erecta*, *D*. *grimshawi*, *D*. *melanogaster*, *D*. *mojavensis*, *D*. *persimilis*, *D*. *pseudoobscura*, *D*. *sechellia*, *D*. *simulans*, *D*. *virilis*, *D*. *yakuba*) were aligned using the standard settings in *clustalW2*, and analyzed for consecutive instances of perfect nucleotide conservation across all species (consecCons). For the diagram in Fig 2, a vertical line was drawn for at every position in the sequence in which that nucleotide, and the following nucleotide, were identical for all species.

### Western blotting

Ovaries from females raised on yeast for 3–4 days were dissected in ice-cold PBS and prepared as described [29] using ice cold lysis buffer (25mM Tris-Cl pH6.8, 1mM MgCl_2_, 100mM KCl, 1mM DTT, and 0.1% Triton X-100). Lysates were run on a 7% SDS-PAGE gel and transferred to PVDF membrane. Antibodies were used at the following dilutions: mouse anti-HA (1:1000, Covance), mouse anti- α-tubulin (1:2000, Sigma), and mouse anti-GFP (1:1000, Santa Cruz). Secondary antibodies were alkaline phosphatase-conjugated goat anti-mouse IgG (Applied Biosystem)(1:5,000). Signals were detected using CDP-Star chemiluminescent substrate (Sigma) and exposure to X-ray film (Kodak BioMax).

### Whole mount immunodetection

Sample preparation, antibody staining, and microscopy were performed as described [5,6]. Quantitations were performed on samples fixed, processed and imaged in parallel with identical settings. To estimate levels of protein localized to the posterior pole of oocytes, the fluorescent signal in a single focal plane, judged to be the strongest, was traced and the sum of intensity measured using Fiji. A crescent of similar size and shape was traced at the anterior of the oocyte where Osk is not expressed. The anterior mean intensity was subtracted from that of the posterior. This approach for protein quantitation reliably detects that there are differences, as well as the relative degrees to which mutants are affected. However, it does not provide accurate measurements for absolute amount of Osk protein, because the protein is distributed across a significant *z* dimension in a cap at the posterior of the oocyte and we measure in only a single *z* position. When posterior Osk protein levels are low, the cap of Osk protein shrinks in size leading to both a smaller crescent of protein detected in a single *z* position, as well as a narrower distribution in the *z* dimension. Analyzing a single optical section reliably shows the amount of Osk in that section, but does not consider how many optical sections will have detectable Osk. Consequently, the measurements overestimate the amount of Osk when Osk levels are reduced and the cap of Osk protein is smaller.

### Analysis of RNA

In situ hybridization with ovary samples was performed as described [32]. Fluorescent RNA probes for *osk* and GFP were synthesized using the DIG RNA labeling mix (Roche). Samples were mounted on slides with Vectasheild Mounting Medium (Vector Labs), and imaged with the Leica TCS-SP laser scanning confocal microscope.

For RNase protection assays, RNA was isolated from 3–4 day old females using Tri Reagent-LS (Molecular Research Center) as per the manufacturers instructions followed by phenol/chloroform extraction. Assays were performed using the RPA III Kit (Ambion). Following electrophoresis of products in denaturing gels, signals were detected by phosphorimaging with the Typhoon laser scanner (GE Healthcare) and quantitated using Image J. At least three assays were performed for each transgene.
