# Correction: Translational Activation of *Oskar* mRNA: Reevaluation of the Role and Importance of a 5' Regulatory Element

**DOI:** 10.1371/journal.pone.0131381

**Published:** 2015-06-22

**Authors:** The PLOS ONE Staff

In the title of this article, “*Oskar”* should be “*oskar*.” The correct title is: Translational Activation of *oskar* mRNA: Reevaluation of the Role and Importance of a 5' Regulatory Element. The correct citation is: Kanke M, Macdonald PM (2015) Translational Activation of *oskar* mRNA: Reevaluation of the Role and Importance of a 5' Regulatory Element. PLoS ONE 10(5): e0125849. doi:10.1371/journal.pone.0125849

The publisher apologizes for this error.
